# Nuclear Cardiology in the Era of Precision Medicine: Tailoring Treatment to the Individual Patient

**DOI:** 10.7759/cureus.58960

**Published:** 2024-04-24

**Authors:** Biruk D Ayalew, Zarin Nudar Rodoshi, Vaishvik K Patel, Alaa Alresheq, Hisham M Babu, Raja Faizan Aurangzeb, Raja Irsalan Aurangzeb, Marika Mdivnishvili, Abdur Rehman, Abdullah Shehryar, Ahmad Hassan

**Affiliations:** 1 Internal Medicine, Saint Paul's Hospital Millennium Medical College, Addis Ababa, ETH; 2 Medical School, Mymensingh Medical College, Dhaka, BGD; 3 Medical School, St. George's University, West Indies, GRD; 4 Primary Care, United Nations for Relief and Works Agency, Ramallah, PSE; 5 Internal Medicine, Jagadguru Sri Shivarathreeshwara (JSS) Medical College and Hospital, JSS Academy of Higher Education and Research (JSSAHER), Mysore, IND; 6 Internal Medicine, Fauji Foundation Hospital, Rawalpindi, PAK; 7 Cardiac/Thoracic/Vascular Surgery, Jerarsi Hospital, Tbilisi, GEO; 8 Surgery, Mayo Hospital, Lahore, PAK; 9 Internal Medicine, Allama Iqbal Medical College, Lahore, PAK; 10 Internal Medicine, Mayo Hospital, Lahore, PAK

**Keywords:** artificial intelligence, molecular imaging, personalized treatment, cardiovascular diseases, spect, pet, precision medicine, nuclear cardiology

## Abstract

Nuclear cardiology, employing advanced imaging technologies like positron emission tomography (PET) and single photon emission computed tomography (SPECT), is instrumental in diagnosing, risk stratifying, and managing heart diseases. Concurrently, precision medicine advocates for treatments tailored to each patient's genetic, environmental, and lifestyle specificities, promising a revolution in personalized cardiovascular care. This review explores the synergy between nuclear cardiology and precision medicine, highlighting advancements, potential enhancements in patient outcomes, and the challenges and opportunities of this integration.

We examined the evolution of nuclear cardiology technologies, including PET and SPECT, and their role in cardiovascular diagnostics. We also delved into the principles of precision medicine, focusing on genetic and molecular profiling, data analytics, and individualized treatment strategies. The integration of these domains aims to optimize diagnostic accuracy, therapeutic interventions, and prognostic evaluations in cardiovascular care.

Advancements in molecular imaging and the application of artificial intelligence in nuclear cardiology have significantly improved the precision of diagnostics and treatment plans. The adoption of precision medicine principles in nuclear cardiology enables the customization of patient care, leveraging genetic information and biomarkers for enhanced therapeutic outcomes. However, challenges such as data integration, accessibility, cost, and the need for specialized expertise persist.

The confluence of nuclear cardiology and precision medicine offers a promising pathway toward revolutionizing cardiovascular healthcare, providing more accurate, effective, and personalized patient care. Addressing existing challenges and fostering interdisciplinary collaboration is crucial for realizing the full potential of this integration in improving patient outcomes.

## Introduction and background

In the evolving landscape of cardiovascular medicine, nuclear cardiology has emerged as a cornerstone for the diagnosis, risk stratification, and management of heart disease. Utilizing sophisticated imaging technologies, such as positron emission tomography (PET) and single photon emission computed tomography (SPECT), nuclear cardiology offers unparalleled insights into myocardial perfusion, function, and metabolism. Parallel to these advancements, the paradigm of precision medicine has begun to reshape healthcare, advocating for bespoke treatment plans that reflect each patient's genetic, environmental, and lifestyle uniqueness. This narrative review aims to explore the confluence of nuclear cardiology and precision medicine, emphasizing how this integration is poised to revolutionize patient-specific cardiovascular care [1].

The concept of precision medicine, characterized by tailoring medical treatment to each patient's individual characteristics, has gained traction across various medical disciplines, including cardiology. It represents a departure from the traditional "one-size-fits-all" approach, focusing instead on customizing patient care based on genetic information, biomarkers, and other distinctive factors. In nuclear cardiology, this approach promises to refine diagnostic accuracy, optimize therapeutic interventions, and enhance prognostic evaluations, thereby improving clinical outcomes for patients with cardiovascular diseases [2].

Recent advancements in molecular imaging, alongside the integration of artificial intelligence and machine learning algorithms, enable more precise and predictive models of heart disease. These innovations facilitate a more nuanced understanding of cardiovascular pathologies at an individual level, fostering a more strategic and personalized approach to treatment planning [3]. Nuclear cardiology is at the forefront of a medical revolution by bridging cutting-edge imaging techniques with the principles of precision medicine. This review will dissect the current state of this integration, highlight its potential to enhance patient outcomes, and discuss the challenges and opportunities that lie ahead.

## Review

Background

Overview of Nuclear Cardiology

Nuclear cardiology, a pivotal branch of cardiovascular imaging, has significantly evolved since its inception in the mid-20th century. This specialization employs radioactive substances, known as radiotracers, to visualize the heart and vascular system, providing critical information on the structure and function of the myocardium. The genesis of nuclear cardiology can be traced back to the development of the gamma camera in the 1950s, which revolutionized the ability to detect and analyze radiopharmaceuticals within the body. Over the decades, advancements in imaging technology and radiotracer chemistry have expanded the capabilities and applications of nuclear cardiology, making it an indispensable tool in diagnosing and managing heart disease [4].

PET: It is a sophisticated imaging modality that offers superior spatial and temporal resolution compared to other nuclear medicine techniques. PET detects the gamma rays emitted by a positron-emitting radiotracer injected into the body. This technology precisely measures metabolic and physiological processes within the heart, such as myocardial blood flow and viability. PET's ability to quantitatively assess myocardial perfusion and metabolism has made it a gold standard in evaluating coronary artery disease (CAD) and cardiomyopathies [5].

SPECT: It is another cornerstone of nuclear cardiology and utilizes gamma-emitting radiotracers to create three-dimensional images of the heart. SPECT imaging is particularly valuable for assessing myocardial perfusion, enabling the detection of ischemia and infarcted tissue. Beyond these applications, SPECT can also assess myocardial viability using Tl201 or modified protocols of standard perfusion tracers. Additionally, it employs other tracers for evaluating myocardial innervation, detecting cardiac conditions like amyloidosis and sarcoidosis, and diagnosing infections in native or prosthetic valves or endocarditis. This technique has been widely adopted for the risk stratification and management of patients with CAD, offering insights into the extent and severity of the disease [6].

Current State and Advancements

The current landscape of nuclear cardiology is characterized by rapid technological advancements and the integration of new imaging agents, which have enhanced the diagnostic and prognostic capabilities of PET and SPECT. The development of hybrid imaging systems, such as PET/CT and SPECT/CT, combines anatomical and functional imaging, providing a more comprehensive assessment of cardiovascular disease. Additionally, the advent of novel radiotracers targeting specific molecular pathways offers the potential for the early detection of cardiovascular pathologies at a cellular or molecular level [7].

Parallel to these technological advancements, the field of nuclear cardiology is embracing the principles of precision medicine. Despite these advancements, challenges remain in the widespread adoption of advanced nuclear cardiology techniques, including cost, accessibility, and the need for specialized expertise. However, the ongoing research and development in this field continue to address these barriers, aiming to make precision cardiovascular imaging a reality for patients worldwide [8].

Principles of Precision Medicine

Precision medicine represents a transformative approach to healthcare, moving beyond the traditional "one-size-fits-all" treatment paradigm towards more customized care. At its core, precision medicine is grounded in the understanding that individuals vary significantly in their genes, environments, and lifestyles, influencing health and disease outcomes. This approach seeks to leverage these differences, utilizing advanced technologies and data analysis to tailor prevention, diagnosis, and treatment strategies to individual patients [9].

Genetic and molecular information: Precision medicine heavily relies on genetic and molecular profiling to identify the biological underpinnings of diseases in individual patients. By understanding specific genetic mutations or biomarkers, clinicians can predict the disease risk, diagnose conditions more accurately, and select treatments more likely to be effective for the particular patient [10].

Data integration and analytics: Integrating clinical data, including imaging and laboratory results, with genetic information and environmental exposure data, is essential for developing personalized treatment plans. Artificial intelligence and machine learning algorithms are crucial in analyzing this vast data, identifying patterns, and predicting individual therapy responses [11].

Individualized treatment strategies: The ultimate goal of precision medicine is to optimize treatment efficacy and minimize side effects by selecting therapies specifically designed or chosen based on the patient's genetic makeup or the molecular characteristics of their disease. This approach can range from selecting the right drug at the right dose to more advanced therapies, such as targeted gene editing or immunotherapies [12].

Potential for Individualized Treatment Strategies

The potential of precision medicine is vast, offering the promise of highly individualized treatment strategies that are more effective and have fewer side effects. In oncology, for example, precision medicine has already led to significant advancements, with targeted therapies designed to attack cancer cells based on their specific genetic mutations, sparing healthy cells and reducing side effects. Similarly, in cardiovascular disease, precision medicine is beginning to inform the selection of therapies based on genetic risk factors and biomarkers, enhancing the effectiveness of treatments [13,14].

Integration of precision medicine in nuclear cardiology

Integrating precision medicine into nuclear cardiology marks a pivotal shift towards highly personalized cardiovascular care. By harnessing genetic insights and individual risk factors, this approach allows for customizing imaging protocols and optimizing diagnostic and therapeutic precision. Nuclear imaging technologies like PET and SPECT are central to this transformation, offering patient-specific strategies that improve diagnostic accuracy, therapeutic outcomes, and prognostic assessments [15].

Customization begins with genetic profiling, where specific variants inform the selection of radiotracers and imaging protocols, tailoring them to individual metabolic pathways and enhancing the detection and treatment of cardiovascular diseases [16]. Similarly, adjustments for personal risk factors such as diabetes or obesity are made, acknowledging their impact on disease progression and tailoring imaging accordingly to ensure accurate evaluations [17].

Further personalization includes adjusting contrast and radiotracer dosing based on the patient's unique physiological characteristics, reducing radiation exposure, and improving image clarity for better disease management [18]. The stage of cardiovascular disease also influences the choice of imaging techniques, with early and advanced stages requiring distinct approaches for diagnosis and monitoring [19].

Integrating artificial intelligence into nuclear cardiology is not a new concept; it has been a pivotal part of nuclear imaging analysis for years, aiding nuclear medicine physicians in the risk stratification of patients. Artificial intelligence's ability to process and interpret vast amounts of genetic, clinical, and imaging data has historically supported the optimization of imaging protocols and helped in predicting individual patient risks such as radiation exposure or contrast-induced reactions [20]. Today, the field is witnessing significant advancements in artificial intelligence, including the development of more sophisticated algorithms that can provide deeper insights and more accurate predictions based on complex data sets. These advancements are pushing the boundaries of personalized imaging in nuclear cardiology, offering more precise diagnostic accuracy, therapeutic outcomes, and prognostic assessments. This comprehensive, data-driven methodology not only elevates patient care but also underscores the ongoing evolution of cardiovascular diagnostics and treatment, blending established technological innovations with the nuanced needs of the individual. By continuing to integrate these advanced artificial intelligence capabilities, nuclear cardiology can enhance its role in the tailored management of cardiovascular disease, ensuring that patient care keeps pace with technological progress.

Molecular Imaging and Biomarkers in Nuclear Cardiology

Integrating molecular imaging and biomarkers into nuclear cardiology marks a significant step towards achieving the objectives of precision medicine. By identifying and quantifying specific molecular and cellular processes underlying cardiovascular diseases, clinicians gain crucial insights into disease risk, progression, and therapeutic response. This detailed understanding facilitates a more personalized approach to cardiovascular care, allowing for targeted treatment strategies. Molecular imaging in nuclear cardiology leverages advanced technologies such as PET and SPECT, coupled with innovative radiotracers that home in on specific molecular pathways. This approach enables the visualization and quantification of key biological processes critical to the pathogenesis of cardiovascular diseases.

Applications of Molecular Imaging Radiotracers

Myocardial metabolism: For instance, F-18 fluorodeoxyglucose (FDG) is employed in PET imaging to evaluate myocardial metabolism, particularly identifying areas of altered glucose utilization which can indicate ischemic or viable myocardium in patients with CAD [21].

Angiogenesis imaging: Radiotracers such as F-18-labeled RGD peptides target angiogenic processes. These tracers are designed to bind to integrin receptors, which play a crucial role in neovascularization within atherosclerotic plaques or myocardial tissue post-infarction. By highlighting areas of active angiogenesis, these tracers provide valuable insights into plaque stability and the risk of acute coronary events [22].

Cardiac amyloidosis: Pyrophosphate (PYP) in SPECT imaging is specifically used to diagnose and assess the burden of cardiac amyloidosis. This condition involves the deposition of amyloid proteins in the heart tissue, which can lead to heart failure [23].

Neurohormonal activation: Another important tracer is I-123 MIBG (metaiodobenzylguanidine), used in the assessment of cardiac sympathetic nerve activity. This is particularly relevant in the context of heart failure, where altered sympathetic activity is a key feature. MIBG imaging can indicate the degree of neurohormonal activation, which is crucial for prognosis and guiding therapy in heart failure management.

Inflammation imaging: F-18 FDG is also pivotal in imaging inflammation associated with vascular diseases, such as vasculitis or inflamed atherosclerotic plaques, providing insights into the inflammatory status and potential for acute events.

Biomarkers in Nuclear Cardiology

Biomarkers play a crucial role in enhancing the predictive power of nuclear cardiology. They can be derived from blood tests, imaging findings, or genetic testing, offering insights into the molecular mechanisms driving cardiovascular diseases. Biomarkers are invaluable for risk stratification, guiding treatment decisions, and monitoring therapeutic responses. Key applications include the following.

Biomarkers of myocardial injury: Troponins and natriuretic peptides are well-established biomarkers that indicate myocardial injury and stress, respectively. Their levels can guide diagnosing and managing acute coronary syndromes and heart failure [24].

Genetic biomarkers: Genetic markers, such as polymorphisms in genes related to lipid metabolism or clotting pathways, can predict an individual's risk of developing CAD and their potential response to specific therapies, such as statins or anticoagulants [25].

**Figure 1:**
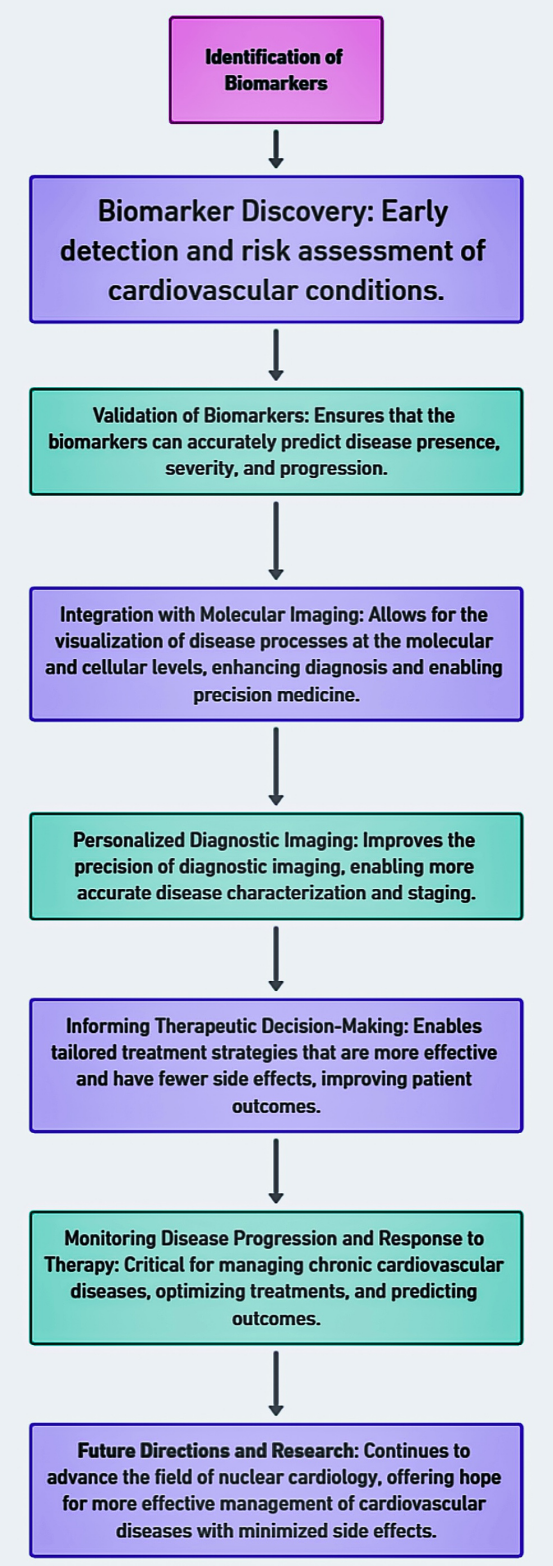
The journey from biomarker identification to advancing cardiovascular disease management. Image Credit: Authors

Imaging-derived biomarkers: Advanced analysis of PET and SPECT images can quantify myocardial blood flow, perfusion defects, and metabolic activity, serving as biomarkers for disease severity and prognosis [26]. An overview of the complete discussion is provided in Figure 1. 

Challenges and opportunities in precision medicine and nuclear cardiology

Precision medicine's integration into nuclear cardiology represents a significant evolution in healthcare, offering tailored patient care through advanced imaging techniques and genetic insights. However, this convergence brings opportunities and challenges that must be navigated to optimize patient outcomes.

Recent enhancements in PET and SPECT technologies have significantly sharpened image resolution, thereby improving myocardial visualization and facilitating early detection of heart diseases [27]. The integration of PET/CT and SPECT/CT into hybrid imaging systems has revolutionized cardiovascular diagnostics, providing detailed anatomical and functional insights that are crucial for accurate diagnosis and effective treatment planning [28]. Additionally, the development of new radiotracers and biomarkers allows for the precise targeting of specific cardiovascular pathologies, directly influencing therapeutic decisions [29]. Complementing these advancements, artificial intelligence and machine learning technologies have become essential in interpreting complex data sets, offering predictions about disease progression and response to treatment that tailor care to individual patient needs [4].

However, integrating precision medicine into clinical practice is fraught with challenges. Data management poses significant hurdles, as does integrating diverse information sources, necessitating sophisticated systems for efficient analysis [30]. The high costs and limited availability of advanced imaging and genetic testing restrict access, highlighting the need for cost-effective solutions and broader accessibility to ensure equitable care. Translating technological advances into routine clinical applications requires validating new imaging agents and artificial intelligence algorithms, training healthcare professionals, and establishing comprehensive guidelines [31]. Additionally, ethical and privacy concerns demand stringent data protection measures to maintain patient trust and uphold ethical standards in healthcare [32].

Conversely, precision medicine in nuclear cardiology opens doors to improved patient outcomes through customized diagnostic and therapeutic approaches. It unveils innovative therapeutic targets by unraveling new molecular pathways in cardiovascular disease. Moreover, it fosters interdisciplinary collaboration, which is crucial for advancing research and translating findings into practice.

Addressing practical challenges is paramount for the successful adoption of precision medicine. Ethical imperatives are ensuring patient privacy, securing informed consent, and preventing genetic discrimination [33,34]. Equitable access to precision medicine remains a concern, necessitating strategies to overcome cost and accessibility barriers and to integrate complex data into clinical decision-making [35-37].

Future directions of nuclear cardiology in precision medicine

The integration of precision medicine into nuclear cardiology is poised for significant growth and innovation, promising to revolutionize the diagnosis, treatment, and management of cardiovascular diseases. As we look to the future, several areas are particularly ripe for development, including new therapies, diagnostic tools, and approaches that leverage the latest technological advancements [38]. The phenotypic variations among individuals, such as in chronic heart failure patients, will lead to the integration of genetics in management surpassing the simple stratification based on the variable of interest, such as their cholesterol level, blood pressure, or history of myocardial infarction, but rather on their complex phenotype.

Advanced Imaging Technologies

The future of nuclear cardiology is poised to benefit from groundbreaking advancements in imaging technologies. Upcoming developments include quantum imaging techniques, which promise to revolutionize the field by enabling exceptionally high-resolution images with significantly faster acquisition times, thereby reducing radiation exposure for patients. Another notable innovation is the use of photon-counting detectors, which offer superior image quality by accurately counting individual photons. This technology enhances the sensitivity and specificity of nuclear cardiology procedures, facilitating earlier disease detection and more precise evaluation of treatment efficacy. These technological advancements are set to transform patient diagnostics and care in nuclear cardiology by providing clearer, more accurate imaging results and allowing for real-time monitoring of disease progression and therapeutic response [38].

Next-Generation Radiotracers

Developing novel radiotracers that target specific molecular pathways involved in cardiovascular diseases is an exciting area of research. These tracers could improve our ability to visualize and quantify biological processes at the cellular and molecular levels, from myocardial metabolism and perfusion to inflammation and fibrosis. This precision in imaging could lead to earlier diagnosis and more targeted therapies for conditions such as atherosclerosis, heart failure, and cardiac amyloidosis [39].

Personalized Therapies

Precision medicine's ultimate goal is to tailor treatments to the individual patient, and nuclear cardiology can play a key role in achieving this. Theranostics, which combines diagnostic imaging with targeted therapy, is a promising approach. For example, radiotracers that diagnose and deliver therapeutic agents directly to diseased tissue could offer highly effective treatments with minimal side effects. Additionally, advances in gene editing and regenerative medicine, informed by precise diagnostic imaging, could lead to novel treatments for genetic cardiac conditions and myocardial recovery post-injury [40].

Integration of Artificial Intelligence and Machine Learning

Artificial intelligence and machine learning are expected to become integral to nuclear cardiology, enhancing everything from image acquisition and analysis to risk prediction and treatment planning. AI algorithms can process and interpret vast amounts of imaging data, identifying patterns and correlations that may not be apparent to the human eye. This can lead to more accurate diagnoses, personalized risk assessments, and optimized treatment strategies based on individual patient data [41].

Wearable Technologies and Remote Monitoring

Integrating wearable technologies and remote monitoring devices with nuclear cardiology could transform patient management, particularly for chronic conditions like heart failure. These devices can continuously collect data on vital signs, physical activity, and other health metrics, which can be analyzed in conjunction with imaging data to provide a comprehensive view of a patient's health status and response to treatment [42].

Global Access and Equity

Some experts express skepticism regarding the global accessibility of precision medicine and its impact on cardiovascular medicine, considering it a complex and costly aspect of healthcare with limited benefits. Nevertheless, genetic studies have aided in identifying underlying mutations associated with cardiac diseases. Ongoing research into these mutations, their role in predisposing individuals to cardiac diseases, and strategies for prevention hold significant promise. Therefore, ensuring global access to the benefits of precision medicine in nuclear cardiology emerges as a crucial future direction. Achieving this goal will necessitate concerted efforts to lower costs, enhance the availability of advanced imaging and genetic testing, and devise scalable and sustainable models for delivering personalized care across diverse healthcare settings worldwide [43].

Ethical and Regulatory Frameworks

As the field advances, developing ethical and regulatory frameworks that address privacy, data security, and the equitable use of genetic information will be essential. These frameworks must balance innovation with the protection of patient rights and ensure that the advances in precision medicine benefit all population segments.

**Figure 2:**
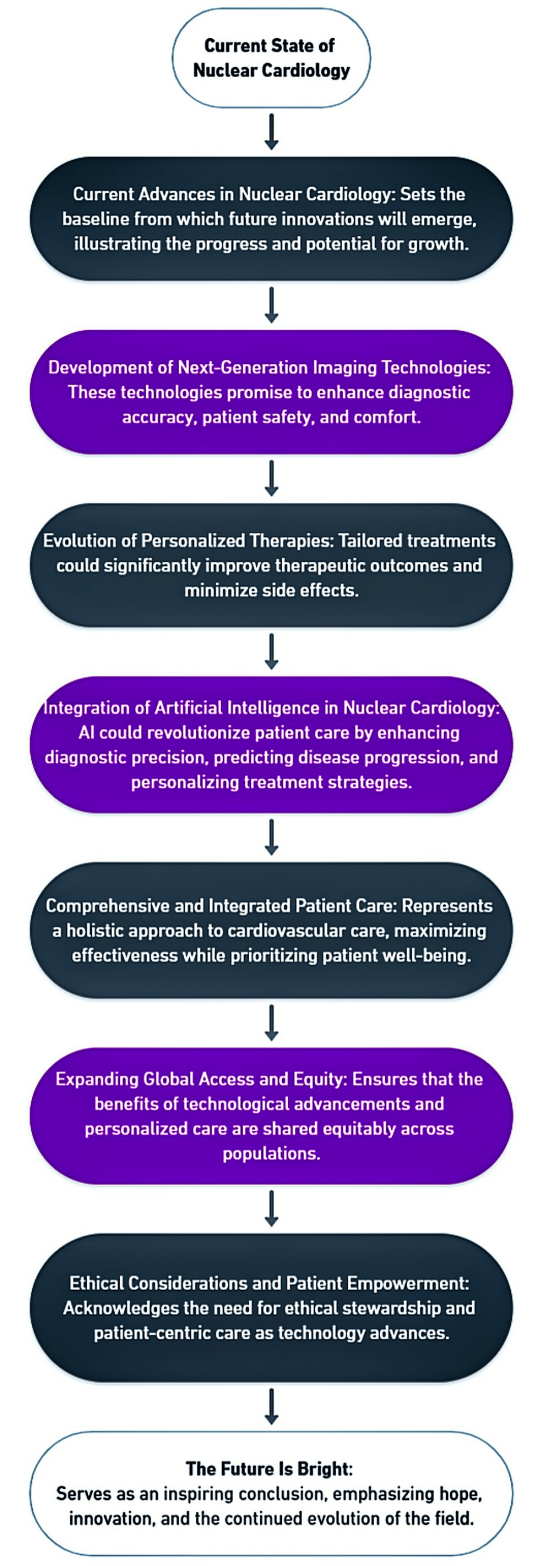
A roadmap for the future of nuclear cardiology. Image Credit: Authors

The future of nuclear cardiology in precision medicine is bright, potentially bringing about a more effective, efficient, and personalized cardiovascular care era. By continuing to invest in research, technology, and infrastructure and addressing the ethical and practical challenges head-on, the field can achieve its full potential to improve patient outcomes and transform cardiovascular health [44]. Figure 2 provides a complete summary of the discussion. 

## Conclusions

In summarizing the critical insights from this review, the integration of precision medicine into nuclear cardiology marks a transformative era in cardiovascular care, shifting from generalized treatments to highly personalized therapeutic strategies. This union capitalizes on the advancements in imaging technologies and genetic profiling to offer refined insights into individual patients' cardiovascular conditions, facilitating tailored diagnostic and therapeutic approaches that promise enhanced outcomes. However, realizing this integration's full potential hinges on overcoming challenges related to data management, accessibility, ethical considerations, and the need for specialized training and validation of new technologies. Addressing these issues is essential for advancing personalized patient care in nuclear cardiology, emphasizing the need for continued interdisciplinary collaboration, innovation, and ethical stewardship. The future of nuclear cardiology in precision medicine is bright, with significant implications for improving patient outcomes and transforming the landscape of cardiovascular healthcare.
